# A Comparison of the Effects of Raw and Processed Amaranth Grain on Laying Hens’ Performance, Egg Physicochemical Properties, Blood Biochemistry and Egg Fatty Acids

**DOI:** 10.3390/ani13081394

**Published:** 2023-04-18

**Authors:** Ruhollah Kianfar, Ambra Rita Di Rosa, Neda Divari, Hossein Janmohammadi, Babak Hosseintabar-Ghasemabad, Marianna Oteri, Ivan Fedorovich Gorlov, Marina Ivanovna Slozhenkina, Aleksandr Anatolievich Mosolov, Alireza Seidavi

**Affiliations:** 1 Department of Animal Science, Faculty of Agriculture, University of Tabriz, Tabriz 51666-16471, Iran; 2 Department of Veterinary Sciences, University of Messina, 98168 Messina, Italy; 3 Volga Region Research Institute of Manufacture and Processing of Meat-and-Milk Production, 400131 Volgograd, Russia; 4 Department of Animal Science, Rasht Branch, Islamic Azad University, Rasht 41335-3516, Iran

**Keywords:** amaranth, blood glucose, health, omega-6, triglyceride, yolk cholesterol

## Abstract

**Simple Summary:**

In ancient civilizations, such as the Maya and Aztecs, amaranth was commonly used in feeding, but due to social and religious changes, it was forgotten. The present research focused on the *Amaranthus hybridus chlorostachys* species. The aim was to investigate the nutritional effects of raw and processed forms of amaranth grain on the performance and health parameters, as well as egg quality traits, in laying hens. The findings of the present research showed that the studied amaranth, while having valuable nutritious and bioactive compounds, can be used in raw and processed forms at low consumption levels (5 and 10%) in the diet and lead to improvements in bird health and the production of low-cholesterol and low-triglyceride eggs without a negative impact on egg weight, feed conversion ratio (FCR) and egg physicochemical properties. However, egg production and egg mass were decreased compared to the control group.

**Abstract:**

In order to investigate the effects of using different levels of either raw or processed amaranth (*Amaranthus hybridus chlorostachys*) grain on performance productivity, egg physicochemical properties, blood biochemistry and egg fatty acids, a trial was conducted using 168 Hy-line W-36 laying hens (67 week of age) in a completely randomized design with seven treatments and six replications of four birds for eight weeks. The trial treatments included the control group receiving no amaranth and the test groups receiving 5, 10 and 15% of raw or autoclaved (120 °C for 5 min) amaranth grain based on dry matter. The results showed that the use of processed amaranth up to the level of five and ten percent of the diet compared to raw amaranth resulted in a better performance than the control group (*p* < 0.05). The consumption of amaranth decreased blood glucose, cholesterol and triglyceride of trial birds without having a negative effect on their health and blood antioxidant status (*p* < 0.05). The use of different forms of amaranth in diets of laying hens had no negative effects on the physicochemical properties of eggs and led to the production of eggs with reduced yolk cholesterol and triglyceride; however, the omega-6 content in eggs and the ratio of omega-6/omega-3 increased (*p* < 0.05). In conclusion, the use of amaranth grain at low levels in the diet of laying hens can enhance the health of the bird and the production of quality and useful eggs.

## 1. Introduction

The poultry industry is one of the most important components of human food security. Therefore, increasing production performance and improving bird health by using new edible plants is one of the goals of poultry nutritionists [1]. In March 1974, in Virginia, USA, a meeting on the world’s underutilized plants was held under the auspices of the National Science Association of Washington National University. One of the highlights from the meeting was that throughout history, humans have consumed approximately three thousand species of plants as food, of which at least 150 species were mass cultivated to some extent in the agricultural system, but over the centuries, the variety and number of edible plants have declined [2,3]. At this meeting scientists from all over focused on 36 edible plants (medicinal plants and tree species were omitted) to prioritize efficient nutritional research to strengthen the food security of the people of the world in accordance with climate change [2]. Later, the World Health Organization (WHO) introduced amaranth as one of the recommended edible plants in the list of neglected and underutilized species (NUS). The plants introduced in the NUS system have been introduced to create a safe production network and to improve economic conditions against agricultural and climatic problems in the world. The literature showed that the use of amaranth in human and livestock nutrition has an important role in improving health and food security [4].

The use of amaranth for consumption was common in the ancient civilizations of the Maya and Aztecs, but due to social and religious developments, it was forgotten. The United States of America began extensive research on amaranth at the Rodale research center in 1970. The Chinese Academy of Agricultural Sciences also conducted much research on agriculture and nutrition from amaranth [5,6]. *Amaranthus*, with a C_4_ photosynthetic system, is a heat and drought-tolerant genus that has 75 species with 11 species growing in Iran [7,8]. Pseudo-cereal amaranth grains are similar true cereals in energy content. Amaranth grains, however, are gluten-free and have nutrients in higher concentrations than in cereal grains. According to the Food and Agriculture Organization (FAO), the biological and qualitative characteristics of its protein, with a score of 74, are equal to animal protein sources [5,9,10,11,12]. The content of lysine, methionine and arginine amino acids is two to three times higher than in cereals [13]. Amaranth contains 48–69% starch and 5–8% oil, levels higher than in other true cereals [5]. Amaranth is also a rich source of phosphorus, magnesium and potassium [5]. The amounts of vitamins in amaranth are generally high. In comparison with wheat, the amounts of riboflavin, vitamin C, folic acid and vitamin E are higher in amaranth, but it is deficient in thiamine [14]. Calcium and phosphorus of amaranth make up about 3.5% of its dry matter [13].

Tillman and Waldroup [15] investigated the extruded amaranth grain of *A. cruentus* species on laying hens at four levels of 0, 10, 20 and 30% and reported that by increasing amaranth levels up to 30%, egg production increased and FCR was reduced compared to the control group. The level of 20% was the practical suggestion of the researchers. Punita and Chaturvedi [16] showed that use at levels of 25% of raw and processed amaranth in the diet of laying hens can effectively reduce fat and cholesterol and increase linoleic acid in egg yolk. Bartkowiak et al. [17] reported that the use of amaranth grain at levels of 2, 5 and 10% leads to a reduction in egg cholesterol. Popiela et al. [18], by feeding extruded amaranth grain at levels of 0, 5 and 10% with 60 Lohmann brown laying hens, showed that the use of 5% amaranth led to an increase in performance and no negative effect on the quality of the eggs. Hosseintabar-Ghasemabad et al. [19] hypothesized that amaranth, as a valuable edible plant source, has potential to meet the nutritional requirements of poultry, and their research findings showed that feeding-processed amaranth to laying hens can lead to the production of low-cholesterol eggs without any negative effect on egg quality. It also improved the antioxidant status and atherogenic index (LDL/HDL) of egg-laying birds. In addition, feeding amaranth with enzyme additives helped to improve production performance. Janmohammadi et al. [1] hypothesized that raw amaranth grain is a rich source of phytosterol, tocopherol and squalene bioactive compounds and its feeding in laying hens at a level of 10% with a multi-enzyme additive, while maintaining production performance, can lead to the improvement of blood, antioxidant parameters and the health of the animal and, parallel with that, the production of healthier, low-cholesterol eggs.

Today, amaranth is receiving the attention of the world as a product with high potential and multi-purpose use. Due to its high resistance to environmental stress, amaranth can be produced more cheaply than any other grain in modern agricultural systems. *Amaranthus hybridus chlorostachys* species seems to have a good potential in terms of distribution and ecological compatibility in many regions and countries, as well as its favorable food quality in nutrition programs. On the other hand, research on this species in poultry nutrition is very limited and the need to know the effects of this unusual food source on the performance and health of laying hens and the quality traits of the produced eggs can be an innovative and important achievement in the poultry industry and, subsequently, food security in the future. Therefore, the aim of the current research is to investigate the nutritional effects of raw and processed forms of amaranth grain on laying hens to evaluate performance, blood biochemistry and assessment of cholesterol, triglycerides and eggs’ fatty acids.

## 2. Materials and Methods

All procedures and efforts used in this study were approved by the Ethics, Care and Use Committee of Animals at Tabriz University.

### 2.1. Birds and Assay Diets

The experiment involved a total of 168 Hy-line W-36 white leghorn laying hens with an age of 67 weeks at the beginning of the experiment, in a completely randomized design with seven treatments and six replications with four birds per replicate in each cage (41 cm × 22 cm × 43 cm). Each cage was considered an experimental unit. The research was conducted in the laying farm of Khalat Pooshan research station of Tabriz University (Tabriz-Iran).

The trial treatments included the control group, three levels of raw amaranth grain (ARaw) (5, 10, and 15%) and three levels of processed amaranth grain (A120°C) (5, 10, and 15%) based on dry matter. The trial diets were formulated based on linear programming by User-Friendly Feed Formulation Done Again (UFFDA) software [19] and according to the catalogue of leghorn hen’s requirements (Table 1).

The experiment ran for 8 weeks (adaptation 2 wk. + main experiment 6 wk.), following the recommendation of the Hy-line W-36 white leghorn guide for breeding standards for (1) the amount of feed offered (FI), (2) the lighting regime and (3) the temperature control during the entire experiment. Before starting the experiment, laying hens whose egg production was equal and close to each other was placed in each replicate. In addition, the body weight of laying hens was recorded at the beginning and end of the period, as well as the amounts of losses during the period. Access to drinking water was *ad libitum* and the amount of daily diet was provided to the birds based on the recommendations of the leghorn hens’ catalog.

### 2.2. Test Ingredients

*Amaranthus hybridus chlorostachys* used in this research, which is cultivated in the south of the Caspian Sea, was obtained from Darvash Giah Khazar medicinal herbs complex company (Ltf) (Rasht, Iran).ARawwere ground using a stone mill (ML218, Ghorbani Electrical Machines Co., Ltd., Rasht, Iran) after the A120°C samples were autoclaved (wet heat of 120 °C for 5 min) [20]. In addition to the availability of analysis of chemical compounds and apparent metabolizable energy modified for nitrogen (AME_n_) of amaranth grain from previous studies and research [21,22], the researchers of the present study re-analyzed the amaranth grain for chemical composition and bioactive phytochemicals. To analyze the chemical composition of amaranth grain, the recommended methods of the Official Analytical Chemist Association (AOAC) [23] were followed. Gross energy (GE) was quantified using a Parr adiabatic bomb calorimeter (Parr 6100 Instruments Co., Ltd., Moline, IL, USA) and benzoic acid as a calibration standard. The Foss 2300 Kjeletc analyzer (ID-number: 020182, Burladingen, Germany) was also used to determine the raw nitrogen values, and by multiplying the nitrogen value by 6.25, the crude protein values were calculated. Next, ether extract (EE) by extraction solvent (Ser 148, VelpScientifica analytical instrument, Usmate, Italy), ash by Shin Seang electric furnace (model: SEF-201, South Korea), crude fiber (CF) by Fibertec (Ser 8000, Foss Analytic, Höganaös, Sweden), non-detergent fiber (NDF) and acid detergent fiber (ADF) by Van Soest et al. [24] were all carried out in the Advanced Animal Nutrition Laboratory of University of Tabriz, Iran.

In the “Specialized Food Laboratory Tekno Azma” in Tehran, Iran, which is approved by the Food and Drug Administration of Iran, the values of linoleic acid (LA) and squalene [25] were determined usinga GC device (Model: Younglin 6100, Anyang, Republic of Korea). Next, tocopherol content was determined by HPLC (Model: Younglin Acme 9000, Anyang, Republic of Korea), while a Jasco FP-4025 detector made in Vienna, Austria based on AOCS [26] was used to measure phytosterols [27,28] (Table 2).

### 2.3. Performanceon Laying Hens

Feed intake, egg weight, egg mass, feed conversion ratio (FCR) and losses were measured and calculated daily on a per pen basis. Egg production was reported as hen day production by calculating the total number of eggs collected divided by the total number of live hens per day, and the FCR was estimated by dividing the feed intake by egg mass.

### 2.4. Egg Physicochemical Properties

At the end of the trial, all the eggs were transferred to the Advanced Animal Nutrition Laboratory of Tabriz University and some quality traits of the eggs, including shell thickness and Haughunits, were evaluated and calculated. Haugh units were obtained by the following formula:

Haugh unit = 100 log HA + 7.57 − 1.7 EW^0.37^, where HA and EW represent the egg height and egg weight, respectively. Shell thickness was measured using a micrometer from three different parts of the egg [29].

It should be noted that the number and the weight of eggs were measured every day (to evaluate performance). However, for egg quality traits, sampling was carried out only at the end of the period.

### 2.5. Egg Yolk Cholesterol, Triglyceride and Fatty Acid Content

To measure the above-mentioned parameters, two eggs per cage were randomly selected at the end of the period and, after weighing, they were transferred to the Medical Sciences Service Laboratory of Tabriz University (Pashmina). In the laboratory, the egg white was separated from the yolk and weighed separately, before the yolk was mixed and homogenized. Then, one gram of the yolk sample was mixed with 50 mL of sodium hydroxide and 50 mL of hydrochloric acid for 10 min and then centrifuged at 3000 rpm at 20 °C [30,31,32].

According to the protocol and report of Baghban-Kanani et al. [33], one gram of yolk was homogenized with 15 mL of chloroform–methanol in a ratio of 2:1 by volume. It was sonicated and filtered, and egg cholesterol and egg triglyceride were determined. To determine the profile of fatty acids, all yolk samples were obtained following the procedure by Folch et al. [34], and fat was extracted following the procedure by Metcalfe and Schmitz [35]. In the chloroform phase, the samples were filtered by filter paper. After centrifugation and smoothing of the samples, one microliter of the upper phase was injected into a 2001 model gas chromatograph, Agilent, Palo Alto, CS, USA, and an HP5MS column at 180 °C for 10 min. The device program was set and started and increased to 195 °C at 5 °C/min. Injector and detector temperature was set to 230 °C, and helium was used as the carrier gas at a flow rate of 1.5 mL/min [36].

### 2.6. Blood Biochemistry Parameters and AntioxidantStatus

At the end of the trial, one bird was randomly selected from each cage and blood was collected from the wing vein using five-milliliter syringes. After blood collection, all plasma samples were centrifuged for 10 min at a speed of 3000 rpm and they were kept at −80 degrees Celsius. In order to measure the triglyceride and cholesterol levels of blood samples, first the samples were separated in Pars azmoon commercial diagnostic kits, and then they were measured by colorimetric and photometric enzymatic methods, as suggested by Baghban-Kanani et al. [37], Nobakht et al. [38] and Feshanghchi et al. [39]. Glucose was measured using a hexokinase enzyme method according to the proposed methods by Kunst [40], Hosseintabar et al. [41], Li et al. [42] and Mokhtarzadeh [43].

Also, two milliliters of blood were drained into heparin tubes and blood antioxidant analysis was performed according to the method proposed by Hosseini-Vashan et al. [44]. A Randox commercial kit (Crumlin, Great Britain) was used to measure the total antioxidant capacity (TAC) and malondialdehyde (MDA) status of the blood [44]. Using the instructions suggested by Kei [45], Yagi [46] and Wang et al. [47], 1,1,3,3-tetraethoxypropane was reacted with thiobarbituric acid (TBA) after extraction with isobutanol. The values of these two parameters were obtained using a spectrophotometer (Shimadzu V-1201 model (Kyoto, Japan) at a wavelength of 532 nm [32]. All the activities of this part of the experiment were carried out in the Medical Sciences Laboratory of Tabriz University.

### 2.7. Statistical Analysis

After homogeneity tests of variance and normality tests for the residuals, the data for all groups except the control were analyzed as a 2 × 3 factorial with two amaranth types, namely raw (ARaw) and processed (A120), and with three amaranth levels (5, 10 and 15%). A single orthogonal contrast was used to determine the comparison of means between the control vs. all amaranth groups combined. SAS 9.3 statistical software was used for all statistical tests.

## 3. Results

The results for the performance parameters for the fixed effects of amaranth type, amaranth level, the interaction effects of amaranth type and level, and the orthogonal comparison between the amaranth groups and the control treatment are presented in Table 3. The results show that the use of processed amaranth significantly increased egg production, feed intake and egg mass compared to raw amaranth grain (*p* < 0.001). Furthermore, the FCR value for processed amaranth was significantly lower than that of raw amaranth (*p* < 0.001).

Furthermore, the results showed that amaranth inclusion levels at 5% showed better performance than at other levels (*p* < 0.001). Levels of 5 and 10% amaranth showed better performance in terms of egg production, feed intake and egg mass than the level of 15%. Investigating the interaction effects of amaranth type and amaranth levels on functional parameters showed that processed amaranth at 5% inclusion level had better performance in terms of egg production, feed intake and egg mass than other treatments.

For egg production, feed intake and egg mass, raw amaranth at 15% level of inclusion was significantly different from all levels of processed amaranth (*p* < 0.05). Generally, for the performance parameters of egg production, feed intake and egg mass at the corresponding amaranth levels and processed amaranth (5 by 5, 10 by 10 and 15 by 15),performance was higher compared to raw amaranth. Results for the control group showed an increase of 3.24%, 3.59 gr and 2.44 unitsin egg production, feed intake and egg mass, respectively, compared to the amaranth groups (*p* < 0.05). For egg weight and FCR parameters, no significant difference was observed between the control group and amaranth groups (*p* > 0.05). In general, processed amaranth had a better performance compared to raw amaranth, but compared to the control group, performance did not improve significantly.

The results for egg physicochemical properties are presented in Table 4. The results show that the Haugh unit and shell thickness were not affected by the type and levels of amaranth and were similar to the control group (*p* > 0.05). The amount of egg yolk triglycerides in the groups fed with raw amaranth was 0.7 g less than the group fed with processed amaranth (*p* < 0.01).

For egg yolk parameters with increases in amaranth levels, the effect of cholesterol and triglycerides decreased (*p* < 0.01). In general, there were no significant differences between the types of processed amaranth or in different levels of amaranth in terms of yolk cholesterol, while for the raw type, with increasing levels of amaranth, there was a noticeable decrease in yolk cholesterol (*p* < 0.05).

Examining the results of orthogonal comparisons of levels and types of amaranths (all six groups) in comparison with the control group showed that the amount of egg yolk triglyceride in the control group was higher by 5.1 g compared to the amaranth groups (*p* < 0.001).

The results of blood biochemistry for fixed effects of amaranth type, amaranth level, interaction effects of amaranth type and amaranth level and orthogonal comparisons between amaranth groups and control treatment are presented in Table 5. The results of the effect of amaranth type on blood parameters showed that amaranth type has a significant effect on blood glucose, cholesterol and plasma triglycerides (*p* < 0.01). Compared to raw amaranth, processed amaranth had significant reduction effects on blood glucose, cholesterol and triglycerides by 5.25, 4.83, and 2.95 units, respectively (*p* < 0.01). In contrast, amaranth type had no significant effect on TAC and MDA parameters (*p* > 0.05). The results of the effect of amaranth levels on blood parameters showed that increasing amaranth levels led to a significant decrease in blood glucose, cholesterol and triglycerides (*p* < 0.001). For blood parameters (TAC and MDA), no effects were observed at the level of amaranth consumption (*p* > 0.05).

Interaction effects (type of amaranth and levels of amaranth) were effective on blood glucose and triglycerides (*p* < 0.01), so that the highest and lowest levels of blood glucose were observed for raw amaranth at levels 5 (216.44) and 15 (197.07), respectively (*p* < 0.05). These values for blood triglyceride were observed for the raw amaranth type at level 5 (97.75) and the processed amaranth type at level 15 (88.04) (*p* < 0.05). In general, no significant differences were observed between the blood glucose levels of amaranth treatments processed at different levels of amaranth. Meanwhile, with the increase in the level of raw amaranth, the level of glucose in the level of glucose in the blood of birds significantly decreased (*p* < 0.05).

For both types of amaranth (raw and processed), the amount of plasma triglyceride was significantly higher for the 5% level of amaranth than for the 10 and 15% levels. Examining the results of orthogonal comparisons of levels and types of amaranths (all six groups) in comparison with the control group showed that blood glucose, cholesterol and blood plasma triglyceride values for birds fed with amaranth were 37.81, 19.44, 11.61 and 2.44 units lower than the control group, respectively (*p* < 0.001).

The results of fixed effects of amaranth type, amaranth level, interaction effects of amaranth type × amaranth level and orthogonal comparisons between amaranth groups with control treatment on egg fatty acids parameters are presented in Table 6. In general, the main effects of amaranth type, amaranth levels and the interaction effect of amaranth type × amaranth levels on egg fatty acids were not significant (*p >* 0.05).Examining the results of orthogonal comparisons of amaranth levels and types (all 6 groups) compared to the control group showed that birds fed with amaranth group diets led to a significant increase of 2.18 and 2.06 units for total PUFA, n-6 and linoleic acid (18:2, n-6) (*p* < 0.001).

## 4. Discussion

### 4.1. Performance

Jimoh et al [48] reviewed on Amaranthus-Related Research extensively. Rodríguez-Ríos et al. [49] fed autoclaved amaranth at levels of 15, 30 and 45% and concluded that the use of a 15% level of amaranth can lead to maintained production performance, which is in agreement with the present study (the type and level amaranth section). Hosseintabar-Ghasemabad et al. [19] reported that feeding-processed amaranth up to level 20% has no negative effect on performance, and if an enzyme additive is used in diets containing amaranth, it leads to the strengthening of synergistic effects and subsequent improvement in performance, which is consistent with the results of the current research and the differences can be attributed to the enzyme addition.

In another study, Popiela et al. [18] reported that using extruded amaranth grain at levels of 5 and 10% does not make a significant difference to the feed intake of laying hens, and at a 5% level, egg production traitsimproved. They found that at the level of 10%, the percentage of egg production was decreased, which is in line with the results of the present research. In addition, at the level of 5% amaranth grain consumption, an increase in egg weight was reported, which was in contrast to the present report. The type of processed and the differences in type of bird were likely the reasons for some differences, but in general, the effective levels on the performance of this research were consistent with the present report.

Tillman and Waldrop [15] showed that feeding extruded amaranth grain to laying hens at levels of 10, 20 and 30% of dry matter in the diet can lead to a reduction in feed consumption, which is consistent with the results of the present study. The reduction in feed consumption for very high levels of consumption of amaranth in the diet of experimental birds can be due to the increase in fiber in the diet, the increase in non-nutritive substances, the presence of phenolic compounds, saponins and bitter substances, the decrease in palatability and, subsequently, the birds’ decreased desire to consume these type of rations. Furthermore, analyzing the nutrient calculations in Table 2 shows that the control group (with the highest lysine concentration) had the best performance compared to the other treatments. In addition, the responses of birds fed amaranth also seemed to follow the same trend in reducing lysine levels in diets. In addition, at the levels of 10 and 20%, an increase in the percentage of egg production was observed, which is not consistent with the results of the present study. Furthermore, egg weight decreased, which is contrary to the results of the present study, and no effect on egg weight was observed. The aforementioned researchers attributed the reasons for the improvement in egg production and egg weight to the increasing age of the birds and the presence of high linoleic acid in the seeds of amaranth. However, amaranth is considered a rich source of lysine, methionine and linoleic acid, which can be effective factors of productive performance parameters. On the other hand, thermal processing in amaranth leads to ease of digestion through the permeability of the cell walls of the endosperm cortex, subsequently improving the bioavailability of nutrient compounds. In addition, the absence of a significant difference in egg mass parameter was contrary to the present report. In general, the aforementioned researchers hypothesized that up to the 20% level of amaranth feeding can have a positive effect on performance, which is only consistent with the results of the present report for levels of 5 and 10% of the processed form compared to the raw amaranth. Some of the differences can be attributed to differences in the type of thermal processing and surface used and differences in amaranth species.

### 4.2. Egg Physicochemical Properties

Thirty percent of the weight of the egg yolk consists of lipids. Additionally, the main components of this lipid are 65% neutral lipids, 30% phospholipids and 4% cholesterol [50]. Therefore, reducing the amounts of triglycerides and cholesterol in egg yolk through manipulation of the diet of birds can be the best and least dangerous method to produce a beneficial egg for those wishing to reduce triglycerides and cholesterol in their diet. Many studies indicate the role of amaranth nutrition in reducing cholesterol and triglycerides in eggs. Because egg lipids are synthesized in the liver and transported through the blood, any factor that reduces triglycerides and cholesterol in blood can indirectly affect the egg yolk [51].

Amaranth contains nutrients and bioactive substances that may reduce blood cholesterol [52,53]. For example, phytosterols and plant sterols in plant feed sources can reduce cholesterol without side effects [54,55]. Other cholesterol-reducing factors in amaranth include fibers, squalene, tocotrienols and isoprenoid compounds [56]. McNaughton [57] hypothesized that increasing dietary fiber from two to four percent leads to a five to ten percent decrease in egg yolk cholesterol.

Linoleic acid (LA) of amaranth grain can also play an important role in eliminating bile acids and reducing cholesterol. Amaranth grain has been found to contain 500–700 mg/kg of squalene [53,58,59]. Squalene can be one of the key compounds in regulating cholesterol biosynthesis and limiting the production rate of hepatic 3-hydroxy-3-methylglutaryl coenzyme-A (HMG-CoA) reductase [53]. Squalene, if provided in the body through nutrition, can go to the liver with the help of chylomicrons and be used to make steroids and bile acids. Bile acids (cholic and deoxycholic) synthesized from squalene are made in liver cells, which can be combined with glycine and taurine and produce bile salts. By increasing the recycling of bile secretion, a decrease in cholesterol occurs. Considering the amount of bile secretion in laying hens, which is about one milliliter per kilogram of body weight per hour, if the recycling of bile secretion can be increased, decreased cholesterol and triglyceride content can be expected [1,28]. St-Onge et al. [60] hypothesized that feeding amaranth grain and its active compounds stimulates the binding of cholesterol with bile acids and prevents the formation of micelles, while also increasing the fermentation effect on the production of fatty acids. It also shortens the chain, which subsequently leads to an increase in the power of cholesterol-lowering effects. In addition, the activation of 7-alpha hydroxylase causes the formation of bile acids from cholesterol, thereby increasing its excretion. It has been shown that the activation of HMG-CoA reductase enzyme leads to the prevention of cholesterol deposition, and bile salt has a hypocholesterolemia role in the body. However, numerous reports consider the balance between biosynthesis pathways and the self-regulation of cholesterol production from squalene. Tang and Tsao [53] reported that tocopherols in amaranth play a significant role in reducing cholesterol and triglyceride synthesis.

Punita and Chaturvedi [16] were able to reduce yolk cholesterol content by 14% by feeding amaranth up to 25% levels of raw and processed forms in the diet of laying hens, and the reason for this is that providing squalene with amaranth feeding leads to a decrease in 3-hydroxy-3-methylglutaryl coenzyme-A (HMG-CoA) reductase in the liver and a subsequent reduction in cholesterol and triglycerides in egg yolk. Reklewska et al. [61] and Bartkowiak et al. [17] reported that egg yolk cholesterol and triglycerides were reduced by feeding amaranth grain to laying hens. The latest research by Rodríguez-Ríos et al. [45] showed that the consumption of amaranth grain has very high potential for reducing cholesterol and triglycerides in egg yolk. Hosseintabar-Ghasemabad et al. [19] later showed a 10% reduction in egg cholesterol by using amaranth, which is in line with the results of the present research. In a literature review of the role of amaranth in animal nutrition by Peiretti [6], it is mentioned that this edible plant can be used to produce products of animal origin with low cholesterol. The set of reports on the effect of dietary amaranth on cholesterol and triglyceride in egg yolk are all aligned and in agreement with the results of the present study.

### 4.3. Blood Biochemistry

The results of blood glucose reduction characteristics of amaranth-fed birds in the present study were consistent with many hematology and diabetic studies related to blood sugar reduction with amaranth consumption in different animal models. For example, Kamal et al. [62] hypothesized that the presence of amaranth protein hydrolysates (APHs) can play a role as a potential source of the reduction blood glucose, subsequently improving antidiabetic and blood pressure conditions, which indicates the effective role of antidiabetic and antihypertensive peptides in nutrition and functional food formulation. It has been found that amaranth consumption leads to a decrease in the activity of gluconeogenic enzyme glucose-6-phosphatase and fructose 1,6-diphosphatase in the livers of diabetic rats, which can lead to a decrease in blood glucose [63]. In general, amaranth grain can provide a protective agent against fructose-induced obesity and diabetes [64]. A series of well-known specific bioactive compounds, such as tocopherol and tocotrienols in amaranth, have properties that include reducing diabetes [53]. It has been proven that amaranth grain contains dipeptidyl aminopeptidase (DPP IV) inhibitors. DPP IV is a key enzyme responsible for inactivating gastric inhibitory polypeptide (GIP) and glucagon-like peptide-1 (tGLP-1), which is considered an insulin-releasing hormone for endocrine cells in the intestine [65]. Research on the effectiveness of DPP IV to reduce blood sugar in diabetic rats, and to protect these laboratory animals against the negative effects of tGLP-1 and GIP hormones, has been proven by consuming amaranth grain as antihypertensive and antidiabetic peptides. The results indicate that the superior biological activity of this specific protein compound in amaranth grain is significantly superior compared to proteins from animal sources [66]. In recent years, it has been proven that a compound named 20-hydroxyacedizone (20 HE), as a specific bioactive compound in amaranth (148–484 mg), has been able to significantly affect muscle protein synthesis and blood glucose reduction, leading to antidiabetic and anti-obesity properties in laboratory mice [53,67]. In the report and research of Qureshi et al. [68], it was found that supplementing the diet of broiler chickens with the grain of *Amaranthus hypochondriacus* and *Amaranthus cruentus* led to a 10–30% reduction in blood cholesterol. It seems that the nutritious and bioactive compounds of amaranth can limit the activity of HMG-CoA enzymes, increase the endogenous excretion of cholesterol, and at the same time decrease blood cholesterol in chickens consuming amaranth grain. However, the presence of tocotrienols and unsaturated forms of vitamin E has a great effect on cholesterol biosynthesis. Lehmann et al. [69] discussed the presence of tocopherol compounds and tocotrienols, especially the gamma and zeta forms, as the main reason for reducing blood cholesterol. By feeding amaranth grain to hamsters, Mendonça et al. [70] and Soares et al. [71] observed a 48% reduction in cholesterol, which was attributed to the presence of peptides limiting the production of HMG-CoA reductase enzymes. It has been proven that squalene leads to changes in gene expression of key influencing enzymes and increases cholesterol removal, as well as increases bile synthesis and cholesterol excretion [72]. Hood et al. [73] reported that eight vitamin E isomers in amaranth oil can reduce cholesterol and triglyceride levels by 30 and 70%, respectively, in experimental birds. Króliczewska et al. [74] observed a decrease in blood cholesterol and triglycerides by feeding amaranth in laying hens. Popiela et al. [18] found no significant effect on blood lipid profile. However, Longato et al. [75] by feeding amaranth of more than five percent led to an improvement in the blood lipid profile in broilers. Hosseintabar-Ghasemabad et al. [19] concluded in their studies that feeding amaranth led to a decrease in LDL and triglycerides and, in parallel, an increase in HDL, as well as a significant improvement in the atherogenic index and antioxidant status of the blood, which is completely in agreement with the present study. However, many studies have shown that a number of compounds lower blood cholesterol and triglycerides, including crude fiber, tocopherols, phytosterols, linoleic acid and squalene, which are abundant in plant food sources such as amaranth [53], and can lead to cholesterol reduction in different animal models, a finding consistent with the results of the present research on laying hens. Maintaining bird health and the economic life of the laying flock is one of the most important goals of poultry, especially laying hens. Maintaining the antioxidant status of the blood and not causing stress through feeding for laying hens in the cage system can be a potential advantage for edible plant sources, such as amaranth in ration formulation.

Tsao and Tang [53] hypothesized that the presence of amaranthine and isoamaranthine compounds in amaranth grain, by influencing the catabolism of tocopherol, leads to the accumulation of tocopherol in blood and tissue, which in turn may play an important role in maintaining the antioxidant status. Alvarez et al. [76] also hypothesized that the presence of phytosterol bioactive compounds, tocopherols and squalene play a significant role in reducing the risks of oxidative stress and helps to improve the health of consumers. Popiela et al. [18] did not observe any negative effect on some blood antioxidant characteristics and liver enzymes by feeding amaranth in laying hens, which was consistent with the results of the present study. Furthermore, Janmohammadi et al. [1] showed that feeding raw amaranth grain to laying hens can lead to a decrease in blood cholesterol and maintain the antioxidant status of the blood in a normal and stable state, in agreement with the results of the present study.

### 4.4. Egg Fatty Acids

Woods and Fearon [77] hypothesized that the optimal ratio of omega-6 to omega-3 for an egg is 19, and any effort to reduce this ratio can be effective in choosing and improving the health of consumers. The present research, considering the use of food sources rich in omega-6 in an assay diet in addition to the old age of the birds, led to an increase in this ratio, which means that it is necessary to use omega-3 sources in future studies in order to achieve a balance between the ratio of omega-6 to omega-3. Popiela et al. [18] did not observe a significant difference in the profile of egg yolk fatty acids with amaranth feeding, but upon increasing the level of amaranth consumption, they encountered an increase in the ratio of omega-6 to omega-3. Bartkowiak et al. [17] observed an increase in omega-6 and a subsequent increase in this ratio by supplementing the diet of laying hens with amaranth grain. It seems that the output of the present results was consistent with the available reports. The necessity of enriching amaranth-based diets with omega-3 sources is a practical and important suggestion for future research.

## 5. Conclusions

The amaranth used in this research is an annual, non-invasive, native and widely available plant in many parts of the world which, due to its distribution and ecological compatibility in countries with extreme climates, can be a desirable component in layer diets. Due to the presence of nutritious and bioactive compounds in amaranth, the use of raw and processed forms of amaranth at low consumption levels (5 and 10%) leads to an improvement in the health of birds and, in parallel, low-cholesterol and low-triglyceride eggs are produced. In addition, feeding laying hens with processed amaranth yielded better performance compared to raw amaranth. On the other hand, the use of amaranth types compared to the control group did not have a negative effect on egg weight, FCR and egg physicochemical properties, but the decrease in egg production and mass was significant.

## Figures and Tables

**Table 1:** Ingredients and chemical composition of trial diets fed based on dry matter (DM) to Hy-line W-36 white leghorn laying hens.

Treatments							
Items	T_1_: Control	T_2_: 5% (ARaw)	T_3_: 10% (ARaw)	T_4_: 15% (ARaw)	T_5_: 5% (A120°C)	T_6_: 10% (A120°C)	T_7_: 15% (A120°C)
Ingredient (%)							
Corn grain	58.27	54.93	49.59	45.24	54.93	49.59	45.24
Soybean meal	23.31	22.36	21.41	20.47	22.36	21.41	20.47
Amaranth grain	0	5.00	10.00	15.00	5.00	10.00	15.00
Oyster shells	11.30	11.30	11.30	11.30	11.30	11.30	11.30
Soybean oil	3.52	3.19	2.85	2.52	3.19	2.85	2.52
Wheat bran	2.0	2.0	2.0	2.0	2.0	2.0	2.0
Di-calcium phosphate	1.37	1.40	1.44	1.47	1.40	1.44	1.47
Premix Vit ^1^ &Min ^2^	0.6	0.6	0.6	0.6	0.6	0.6	0.6
Salt	0.23	0.23	0.23	0.233	0.23	0.23	0.233
Sodium bicarbonate	0.15	0.15	0.15	0.15	0.15	0.15	0.15
DL- Methionine	0.12	0.12	0.12	0.13	0.12	0.12	0.13
Threonine	0.01	0.01	0.01	0.01	0.01	0.01	0.01
Calculated nutrient content (% except for ME)							
ME (Kcal/kg DM)	2720	2720	2720	2720	2720	2720	2720
Crude protein (%)	15.70	15.70	15.70	15.70	15.70	15.70	15.70
Calcium (%)	4.70	4.70	4.70	4.70	4.70	4.70	4.70
Av. phosphorus (%)	0.40	0.40	0.40	0.40	0.40	0.40	0.40
Methionine (%)	0.38	0.38	0.38	0.38	0.38	0.38	0.38
Met+ cysteine (%)	0.65	0.65	0.65	0.65	0.65	0.65	0.65
Lysine (%)	0.82	0.81	0.79	0.78	0.81	0.79	0.78
Threonine (%)	0.59	0.59	0.59	0.59	0.59	0.59	0.59
Tryptophan (%)	0.18	0.19	0.20	0.21	0.19	0.20	0.21

ARaw: Raw amaranth grain; A120°C: Processed amaranth grain. ^1^ Premix vitamin supplement provides per kilogram of diet: Vitamin A, 9000 IU; Vitamin E, 40 IU; Menadione, 3.0 mg; Vitamin D3, 3000 IU; Riboflavin, 4.0 mg; Capantothenate, 12 mg; Nicotinic acid, 50 mg; Choline 300 mg; Vitamin B_12_, 20 mg; Vitamin B_6_, 0.12 mg; Thiamine, 1.5 mg; Folic acid, 1.00 mg; D-Biotin, 0.10 mg. ^2^ Premix mineral supplement provides per kilogram of diet: Trace mineral (milligrams per kilogram of diet): Mn, 110; Zn, 80; Fe 50; Cu 10; Iodine 1; Se, 0.30; Antioxidant 50.

**Table 2:** Values of chemical compositions and phytosterols, tocopherols and squalene content in raw and processed (120 °C for 5 min) *Amaranthus hybridus chlorostachys*.

Items	Raw Amaranth Grain (ARaw)	Processed Amaranth Grain (A120°C)
Dry Matter (%)	90.6	90.5
Crude Protein (%)	16.7	16.7
Ether Extract (%)	5.5	5.4
Crude fiber (%)	10.8	10.5
NDF (%)	34.17	35.15
ADF (%)	6.25	6.92
Ash (%)	6.4	6.1
Linoleic acid (%)	35.15	35.31
Gross energy (Kcal/kg)	4229	4214
Phytosterols (ppm)	3255.08	3195.10
Tocopherols (ppm)	551.01	531.03
Squalene (ppm)	2174.10	2164.39

**Table 3:** Effect of trials diets on the performance on laying hens.

Items		Egg Production (%)	Egg Weight (g)	Feed Intake (g d^−1^ bird^−1^)	Egg Mass (g d^−1^ bird^−1^)	FCR (kgfeed:kg egg)
Amaranth types						
Processed		73.29 ^a^	61.71	91.01 ^a^	45.23 ^a^	2.01 ^b^
Raw		69.11 ^b^	61.78	89.60 ^b^	42.70 ^b^	2.10 ^a^
SEM		0.20	0.15	0.17	0.18	0.01
*p*-value		0.001	0.75	0.001	0.001	0.001
Amaranth levels (%)						
5		72.47 ^a^	61.74	91.30 ^a^	44.74 ^a^	2.04
10		71.32 ^b^	61.76	90.36 ^b^	44.04 ^a^	2.05
15		69.81 ^c^	61.73	89.26 ^c^	43.10 ^b^	2.07
SEM		0.24	0.19	0.21	0.22	0.01
*p*-value		0.001	0.99	0.001	0.001	0.15
Amaranth types × Amaranth levels (%)						
Processed	5	74.09 ^a^	61.65	91.66 ^a^	45.68 ^a^	2.01
Processed	10	73.56 ^ab^	61.77	90.90 ^ab^	45.44 ^a^	2.00
Processed	15	72.22 ^bc^	61.71	90.47 ^ab^	44.57 ^ab^	2.03
Raw	5	70.85 ^c^	61.83	90.95 ^ab^	43.80 ^bc^	2.08
Raw	10	69.08 ^d^	61.75	89.81 ^b^	42.65 ^cd^	2.11
Raw	15	67.40 ^e^	61.76	88.04 ^c^	41.63 ^d^	2.12
SEM		0.34	0.04	0.29	0.31	0.01
*p*-value		0.04	0.93	0.01	0.20	0.57
Contrast between Control and Amaranths						
Control		74.44 ^a^	61.98	93.90 ^a^	46.14 ^a^	2.04
Amaranths		71.20 ^b^	61.74	90.31 ^b^	43.96 ^b^	2.06
SE^C^		0.97	0.25	0.52	0.63	0.02
SE^A^		0.40	0.10	0.21	0.26	0.01
*p*-value		0.004	0.39	0.001	0.003	0.43
CV		3.32	1.01	1.39	3.51	2.70

SE^C^ and SE^A^ are standard error of control (N = 6 pens) and amaranth (N = 36 pens) groups, respectively. ^a–e^ Means within each column with different superscripts are statistically different (*p* < 0.05).

**Table 4:** Effect of trials diets on the egg physicochemical properties in laying hens.

Items		Haugh	Shell Thickness (mm)	Yolk Cholesterol (mg/g Yolk)	Yolk Triglyceride (mg/g Yolk)
Amaranth types					
Processed		78.86	0.31	11.77	178.71 ^a^
Raw		78.11	0.31	11.79	178.01 ^b^
SEM		0.32	0.00	0.13	0.17
*p*-value		0.11	0.99	0.93	0.006
Amaranth levels					
5		78.70	0.31	12.49 ^a^	179.57 ^a^
10		78.02	0.31	11.67 ^b^	178.51 ^b^
15		78.73	0.32	11.18 ^b^	177.00 ^c^
SEM		0.40	0.00	0.16	0.21
*p*-value		0.36	0.64	0.001	0.001
Amaranth types × Amaranth levels					
Processed	5	79.13	0.32	12.15 ^ab^	179.62
Processed	10	78.84	0.31	11.89 ^ab^	179.08
Processed	15	78.60	0.32	11.29 ^bc^	177.44
Raw	5	78.28	0.31	12.83 ^a^	179.53
Raw	10	77.20	0.32	11.45 ^bc^	177.95
Raw	15	78.85	0.32	11.08 ^c^	176.56
SEM		0.56	0.01	0.22	0.29
*p*-value		0.25	0.09	0.043	0.19
Contrast between Control and Amaranths					
Control		78.15	0.31	14.33	183.46 ^a^
Amaranths		78.48	0.31	11.78	178.36 ^b^
SE^C^		0.57	0.01	0.30	0.58
SE^A^		0.23	0.003	0.12	0.24
*p*-value		0.59	0.33	0.33	0.001
CV		1.79	4.15	6.10	0.80

SE^C^ and SE^A^ are standard errors of control and amaranth groups, respectively. ^a–c^ Means within each column with different superscripts are statistically different (*p* < 0.05).

**Table 5:** Effect of trials diets on the blood biochemistry in laying hens.

Items.		Glucose (mg/dL)	Cholesterol (mg/dL)	Triglyceride (mg/dL)	Total Antioxidant Capacity (U/mL)	Malondialdehyde (nmol/mL)
Amaranth types						
Processed		203.15 ^b^	102.56 ^b^	89.21 ^b^	6.21	4.92
Raw		208.40 ^a^	107.39 ^a^	92.16 ^a^	6.19	5.08
SEM		1.08	0.63	0.27	0.05	0.05
*p*-value		0.002	0.001	0.001	0.74	0.31
Amaranth levels						
5		212.11 ^a^	112.25 ^a^	94.53 ^a^	6.26	5.01
10		206.55 ^b^	101.47 ^b^	88.91 ^b^	6.12	5.02
15		198.66 ^c^	101.22 ^b^	88.62 ^b^	6.22	4.97
SEM		1.33	0.77	0.33	0.06	0.06
*p*-value		0.001	0.001	0.001	0.29	0.84
Amaranth types × Amaranth levels						
Processed	5	207.78 ^bc^	109.27	91.30 ^b^	6.28	4.96
Processed	10	201.42 ^cd^	99.43	88.30 ^c^	6.17	4.85
Processed	15	200.24 ^cd^	98.99	88.04 ^c^	6.19	4.95
Raw	5	216.44 ^a^	115.22	97.75 ^a^	6.24	5.05
Raw	10	211.67 ^ab^	103.50	89.53 ^cd^	6.07	5.20
Raw	15	197.07 ^d^	103.46	89.20 ^c^	6.25	5.00
SEM		1.88	1.09	0.47	0.09	0.09
*p*-value		0.002	0.66	0.001	0.63	0.18
Contrast between Control and Amaranths						
Control		243.58 ^a^	124.42 ^a^	102.30 ^a^	5.18 ^b^	4.95
Amaranths		205.77 ^b^	104.98 ^b^	90.69 ^b^	6.20 ^a^	5.00
SE^C^		3.57	2.43	1.36	0.09	0.09
SE^A^		1.46	0.99	0.56	0.04	5.00
*p*-value		0.001	0.001	0.001	0.001	0.58
CV		4.14	5.53	3.61	3.59	4.35

SE^C^ and SE^A^ are standard error of control and amaranth groups, respectively. ^a–d^ Means within each column with different superscripts are statistically different (*p* < 0.05).

**Table 6:** Effect of trials diets on the egg fatty acids (%) in laying hens.

Items		FA1	FA2	FA3	FA4	TM	FA5	FA6	TP1	FA7	FA8	TP2	FA9	FA9	R (TP1/TP2)
Amaranth types															
Processed		0.39	0.04	21.19	7.63	41.75	2.77	38.97	18.86	15.01	3.85	0.69	0.39	0.30	27.82
Raw		0.40	0.04	21.89	7.68	41.31	2.77	38.54	18.55	14.57	3.97	0.74	0.40	0.33	26.23
SEM		0.01	0.004	0.24	0.18	0.31	0.11	0.28	0.18	0.17	0.04	0.03	0.01	0.03	1.15
*p*-value		0.07	0.92	0.06	0.83	0.33	0.97	0.29	0.22	0.07	0.59	0.31	0.28	0.42	0.3
Amaranth levels															
5		0.39	0.04	21.56	7.49	41.28	2.77	38.51	18.59	14.62	3.97	0.75	0.40	0.35	25.31
10		0.39	0.05	21.30	7.55	41.49	2.76	38.72	18.81	14.93	3.88	0.71	0.39	0.32	27.32
15		0.40	0.05	21.75	7.91	41.82	2.78	39.04	18.71	14.82	3.89	0.68	0.40	0.28	28.44
SEM		0.01	0.01	0.29	0.22	0.38	0.14	0.35	0.22	0.20	0.05	0.04	0.01	0.03	1.41
*p*-value		0.48	0.48	0.56	0.34	0.61	0.99	0.56	0.77	0.56	0.39	0.39	0.52	0.35	0.29
Amaranth types × Amaranth levels															
Processed	5	0.38	0.04	21.26	7.39	41.78	2.76	39.02	18.92	15.03	3.89	0.71	0.40	0.31	26.91
Processed	10	0.39	0.04	21.05	7.48	41.47	2.77	38.70	18.84	15.05	3.79	0.71	0.38	0.34	26.83
Processed	15	0.39	0.05	21.27	8.02	41.99	2.79	39.20	18.82	14.95	3.87	0.65	0.39	0.26	29.70
Raw	5	0.40	0.04	21.87	7.60	40.78	2.78	38.00	18.26	14.22	4.05	0.79	0.41	0.39	23.71
Raw	10	0.39	0.05	21.56	7.63	41.50	2.76	38.75	18.78	14.81	3.97	0.71	0.40	0.31	27.81
Raw	15	0.42	0.05	22.24	7.81	41.65	2.77	38.88	18.60	14.69	3.91	0.71	0.41	0.30	27.17
SEM		0.01	0.01	0.42	0.31	0.54	0.20	0.49	0.31	0.29	0.07	0.05	0.01	0.05	1.99
*p*-value		0.50	0.71	0.84	0.75	0.63	0.99	0.55	0.60	0.53	0.55	0.72	0.68	0.51	0.53
Contrast between Control and Amaranths															
Control		0.41	0.05	22.20	8.06	42.23	2.70	39.53	16.62 ^b^	12.73 ^b^	3.89	0.69	0.41	0.28	25.53
Amaranths		0.39	0.04	21.54	7.65	41.53	2.77	38.76	18.70 ^a^	14.79 ^a^	3.91	0.71	0.40	0.32	27.02
SE^C^		0.01	0.01	0.45	0.29	0.55	0.17	0.51	0.32	0.32	0.07	0.06	0.02	0.05	2.11
SE^A^		0.004	0.003	0.18	0.12	0.22	0.07	0.21	0.13	0.13	0.03	0.02	0.01	0.02	0.86
*p*-value		0.12	0.72	0.18	0.20	0.24	0.71	0.17	0.001	0.001	0.79	0.67	0.48	0.43	0.51
CV		6.40	36.23	5.04	9.26	3.24	15.38	3.22	4.26	5.38	4.27	19.16	11.36	36.36	19.24

SE^C^ and SE^A^ are standard error of control and amaranth groups, respectively. ^a,b^ Means within each column with different superscripts are statistically different (*p* < 0.05). FA: fattyacid; MUFA: monounsaturated fatty acid; PUFA: polyunsaturated fatty acids; n-6: omega-6; n-3: omega-3; and R: ratio. FA1:myristic acid (C14:0); FA2: pentadecanoic acid (C15:0); FA3: palmitic acid (C16:0); FA4: stearic acid (C18:0); TM: total MUFA; FA5: palmitoleic acid (C16:1); FA6: oleic acid (C18:1, n-9); TP1: total PUFA, n-6; FA7: linoleic acid (C18:2, n-6); FA8: arachidonic acid (C20:4, n-6); FA9: docosahexaenoic acid (C22:6, n-3); and R (TP1/TP2): total PUFA, n-6/total PUFA, n-3.

## Data Availability

Data is contained within the article.
